# Supplementary material for: MassCube improves accuracy for metabolomics data processing from raw files to phenotype classifiers
Source: Nat Commun. 2025 Jul 1;16:5487. doi: 10.1038/s41467-025-60640-5 (PMC12216001; doi:10.1038/s41467-025-60640-5)
Supplement: Supplementary file 1 — Supplementary Information [file 41467_2025_60640_MOESM1_ESM.pdf]

MassCube improves accuracy for metabolomics data processing from raw files to phenotype classifiers

Huaxu Yu[1], Jun Ding[2], Tong Shen[1], Min Liu[1], Yuanyue Li[1], Oliver Fiehn[1*]

Affiliations:

[1] West Coast Metabolomics Center, University of California Davis, Davis, CA, 95616, USA

[2] China CAS Key Laboratory of Plant Germplasm Enhancement and Specialty Agriculture, Wuhan Botanical Garden, Chinese Academy of Sciences, Wuhan 430074, PR China

* Corresponding author:

Oliver Fiehn: ofiehn@ucdavis.edu

Outline

Supplementary Figures

Supplementary Figure 1. The MassCube untargeted metabolomics workflow.

Supplementary Figure 2. Peak evaluation criteria in MassCube.

Supplementary Figure 3. Standard-free nonlinear retention time correction in MassCube.

Supplementary Figure 4. Validating normalization of systematic mass spectrometry (MS) signal drift.

Supplementary Figure 5. Application of MassCube's multimodal spectral matching algorithms on the Metabolome Atlas of the Aging Mouse Brain dataset.

Supplementary Figure 6. Classifying annotated metabolic features by MassCube fuzzy search

Supplementary Figure 7. Increased search space for fuzzy search.

Supplementary Figure 8. Fuzzy search in MassCube extends chemical enrichment analysis through compound class annotation.

Supplementary Figure 9. Examples of data visualization in MassCube.

Supplementary Figure 10. Detected failed sample injections by MassCube.

Supplementary Notes

Supplementary Note 1. Metadata management in MassCube

Supplementary Note 2. LC-MS analysis of human plasma samples of Alzheimer’s Disease patients

Supplementary Note 3. Sample preparation workflow and experimental configurations for biological application.

Supplementary Note 4. Failed sample injection detection algorithm

Supplementary Note 5. Outline for source code.

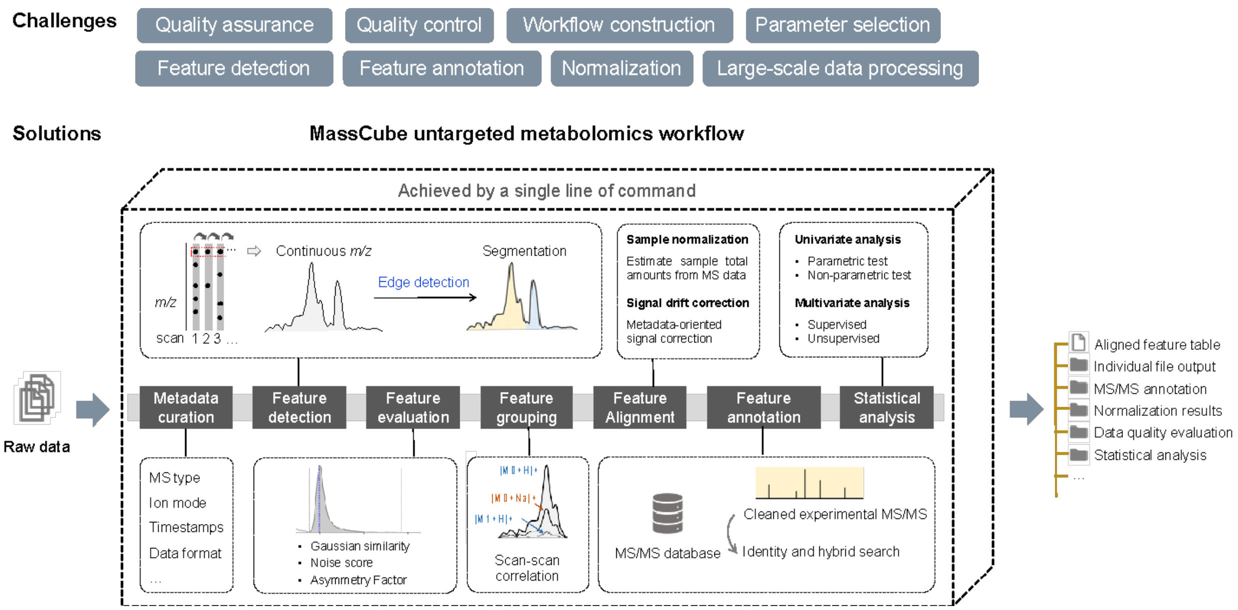

Supplementary Figure 1. The MassCube untargeted metabolomics workflow.
The challenges of performing mass spectrometry data analysis for researchers and the solutions provided by MassCube. Our proposed untargeted metabolomics workflow streamlines the process by offering comprehensive functionalities that can be easily executed by users with no coding experience.

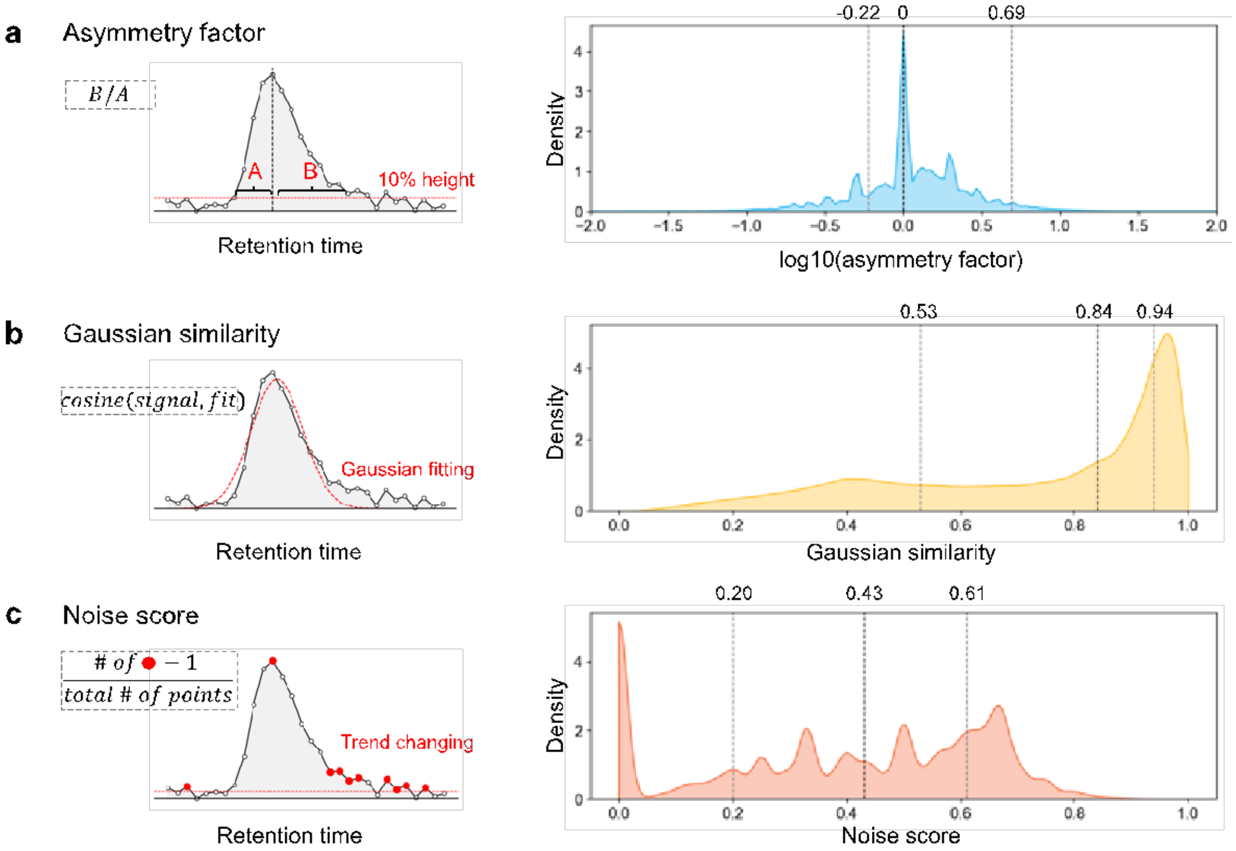

Supplementary Figure 2. Peak evaluation criteria in MassCube.

Asymmetry factor, Gaussian similarity and noise score are calculated in MassCube for peak quality evaluation. Distributions (right panels) of 1,442,223 experimental peaks in 200 files from 41 studies with dashed lines from left to right to represent the 25%, 50%, and 75% quantiles.

(a) Asymmetry factor**.** Left panel: example peak with B/A = 2.75, $\log_{10}$(B/A)=0.44. Right panel: distribution of peak asymmetry factors B/A by log10 over the experimental samples. Red dashed line: 10% peak height.

(b) Gaussian similarity**.** Left panel: example peak with Gaussian fitting. Right panel: distribution of Gaussian similarities over the experimental samples. Red dashed line: Gaussian fitting curve.

(c) Noise score. Left panel, example peak and formula for calculation noise scores from the number of data points and the number of trend changes. Right panel: distribution of noise scores over the experimental samples. Red dashed line: baseline; red dots: MS1 scans indicating a change in intensity trend.

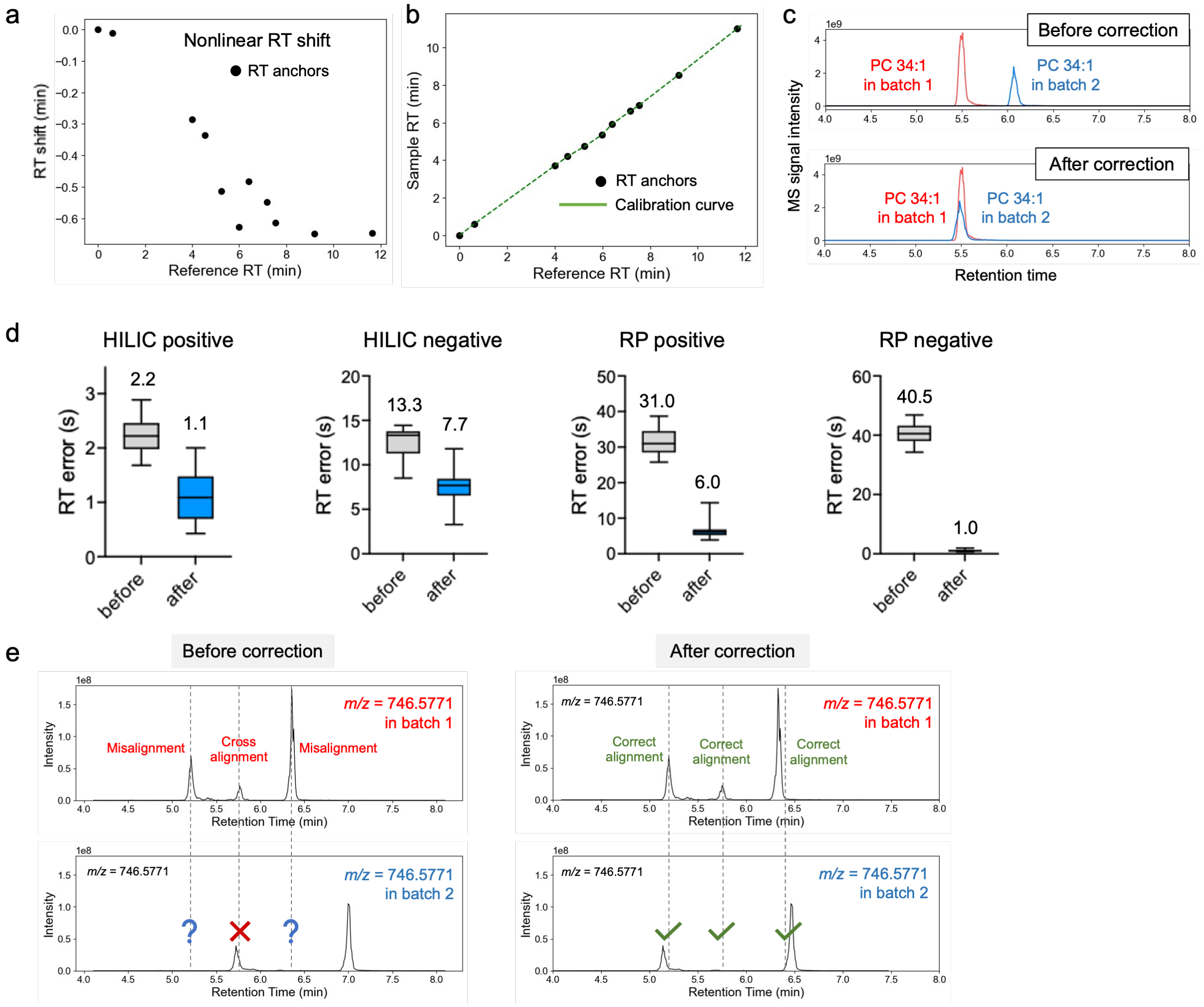

Supplementary Figure 3. **Standard-free nonlinear retention time correction in MassCube**.

(a) Scatter plot shows nonlinear retention shifts across sample groups in the *Metabolome Atlas of the Aging Mouse Brain* dataset, reverse phase positive ion mode. Dots represent the retention time anchors that were automatically selected by MassCube for correction. Anchors represent mass traces with unique peaks found across all samples of a study.

(b) Interpolation model (dashed line) correcting shifts using the retention time anchors.

(c) Validating the RT shift correction using PC 34:1 in reverse phase positive mode as example. Red line: extracted ion chromatogram of PC 34:1 in batch 1; blue line: extracted ion chromatogram of PC 34:1 in batch 2.

(d) Validating the RT shift corrections using 32-38 independent additional RT testing anchors across four metabolomic assays. Whiskers: 5-95 percentile; box: 25-75 percentile with the center indicating median.

(e) Validating the RT shift correction for samples with several peaks for *m/z* mass traces that may cause misalignments in other software programs.

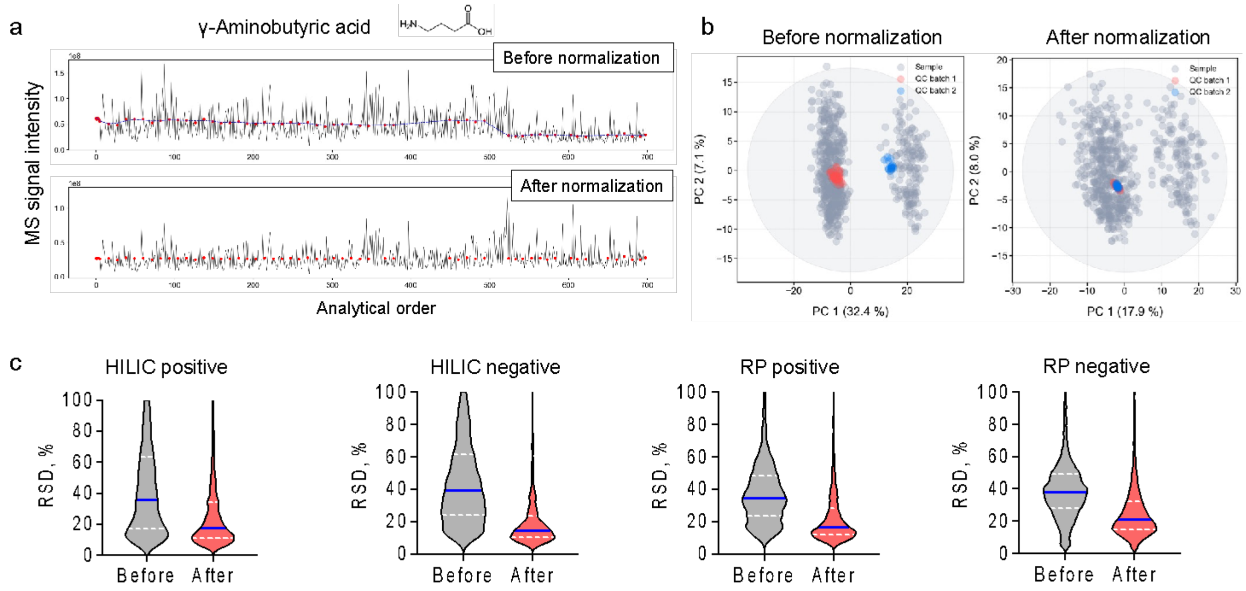

Supplementary Figure 4. **Validating normalization of systematic mass spectrometry (MS) signal drift.**

The *Metabolome Atlas of the Aging Mouse Brain* dataset was used for validation.

(a) An example of the signal drift of the metabolite gamma-aminobutyric acid before and after normalization. The black line represents the MS signal intensity in individual samples plotted by analytical order. Red dots indicate quality control (QC) samples, while the dashed blue line shows the LOWESS calibration curve generated from QC samples. The clear decrease of MS signal intensities before normalization indicates a batch effect.

(b) Principal component analysis results before and after normalization. The clustering of QC samples illustrates the reduced batch effect post-normalization. Data were obtained from the aging mouse brain dataset in case study 1.

(c) Validating LOWESS normalization using QC samples in four ion modes including HILIC positive, HILIC negative, RP positive, and RP negative modes. In each ion mode, a total of 80% QC samples were used as training data, and the rest 20% QC samples were used as testing data. Violin plots show the change of relative standard deviations (RSDs) of testing QCs before and after normalization. Blue line: median value; white dashed lines: 25% and 75% quantiles.

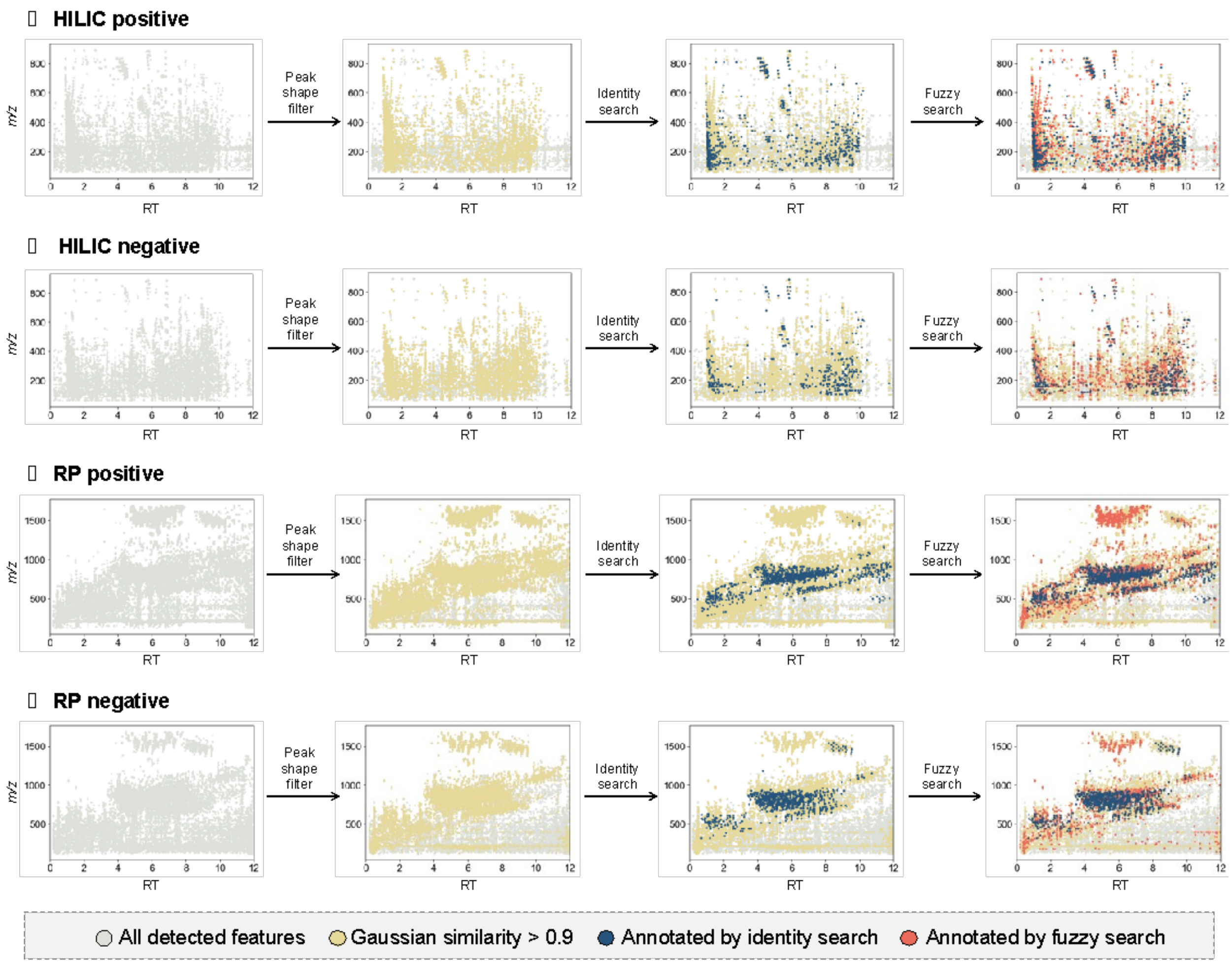

Supplementary Figure 5. Application of MassCube's multimodal spectral matching algorithms on the *Metabolome Atlas of the Aging Mouse Brain* dataset.

Scatter plots show the distribution of peaks across *m/z* and retention time. Panels from top to bottom: peak distribution of HILIC positive, HILIC negative, RP positive and RP negative mode data. For each panel, scatter plots from left to right represent all detected peaks (grey dots), filtered peaks with Gaussian similarity > 0.9 (yellow dots), annotated peaks by identity search (dark blue dots) and annotated peaks by fuzzy search (red dots). Compared to relying only on identity search, fuzzy search in MassCube annotated 1.7- to 3.0-fold more peaks in the respective analytical assays.

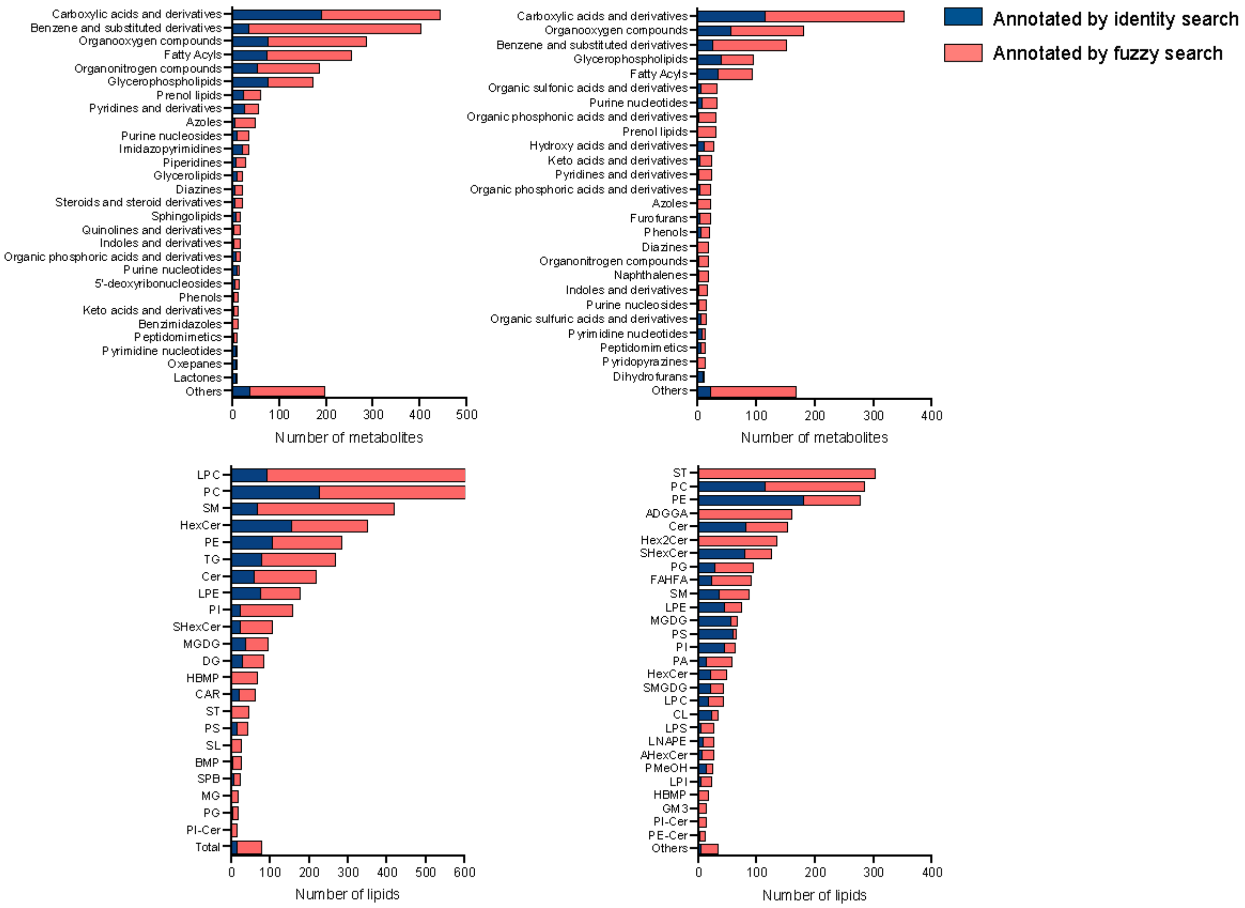

Supplementary Figure 6. Classifying annotated metabolic features by MassCube fuzzy search

Compound annotation in the *Atlas of the Aging Mouse Brain* dataset, stratified into chemical classes using ClassyFire. Top left panel: HILIC positive ion mode; top right panel: HILIC negative ion mode; lower left panel: RPLC positive ion mode; lower right panel: RPLC negative ion mode. Blue: annotated by identity search; red: annotated by fuzzy search.

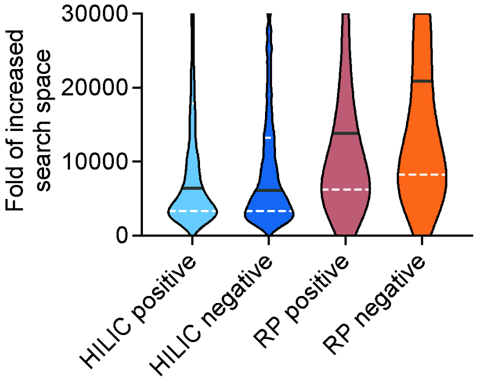

Supplementary Figure 7. **Increased search space for fuzzy search.**

The *Metabolome Atlas of the Aging Mouse Brain* dataset was used to compare the search space expansion in fuzzy search versus conventional identity search. Unlike identity search, fuzzy search imposes no restrictions on precursor m/z during spectral matching, leading to a substantial increase in the number of candidate MS/MS spectra. Violin plots illustrate the significantly expanded search space across all assays, with median fold increases of 6,424, 6,085, 13,844, and 20,857 in HILIC positive, HILIC negative, RP positive, and RP negative modes, respectively. Black lines: median value; white dashed lines: 25% and 75% quantiles.

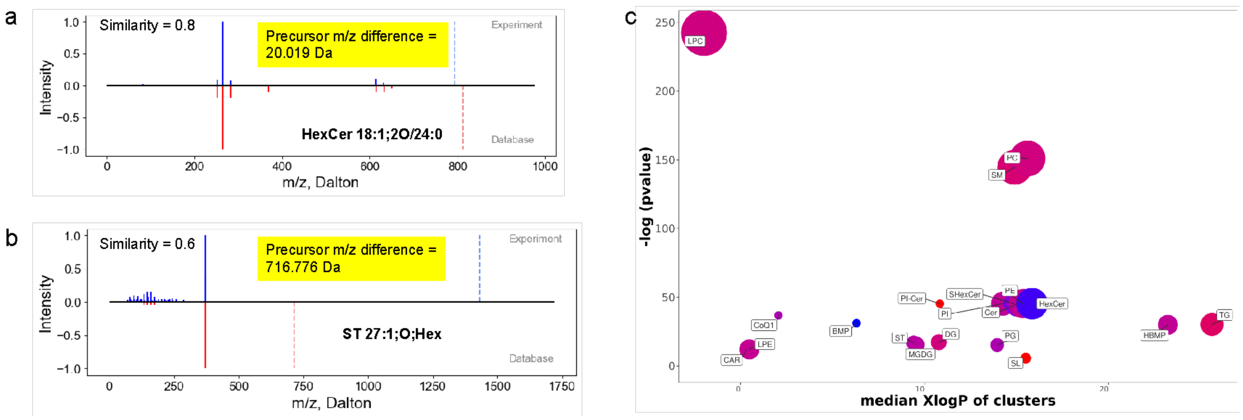

Supplementary Figure 8. **Fuzzy search in MassCube extends chemical enrichment analysis through compound class annotation.**

(a-b) Two examples of annotated hexosylceramide (HexCer) lipid (**a**) and sterol (ST) lipid (**b**) by fuzzy search in RPLC positive ion mode. Solid line: fragment ion; dashed line: precursor ion.

(c) Chemical Similarity Enrichment Analysis (ChemRICH) was performed on annotated lipids to compare the hippocampus region of adolescent (3-week-old) female mice with that of aged (92-week-old) female mice. Circle size is proportional to the number of classified compounds per class. Color scale from blue (all downregulated in 3-week-old mice) to red (all compounds upregulated in 3-week-old mice). Purple colors indicate that specific compounds in the cluster are up-regulated, while others are down-regulated. Statistical enrichment significance is calculated by the Kolmogorov-Smirnov test.

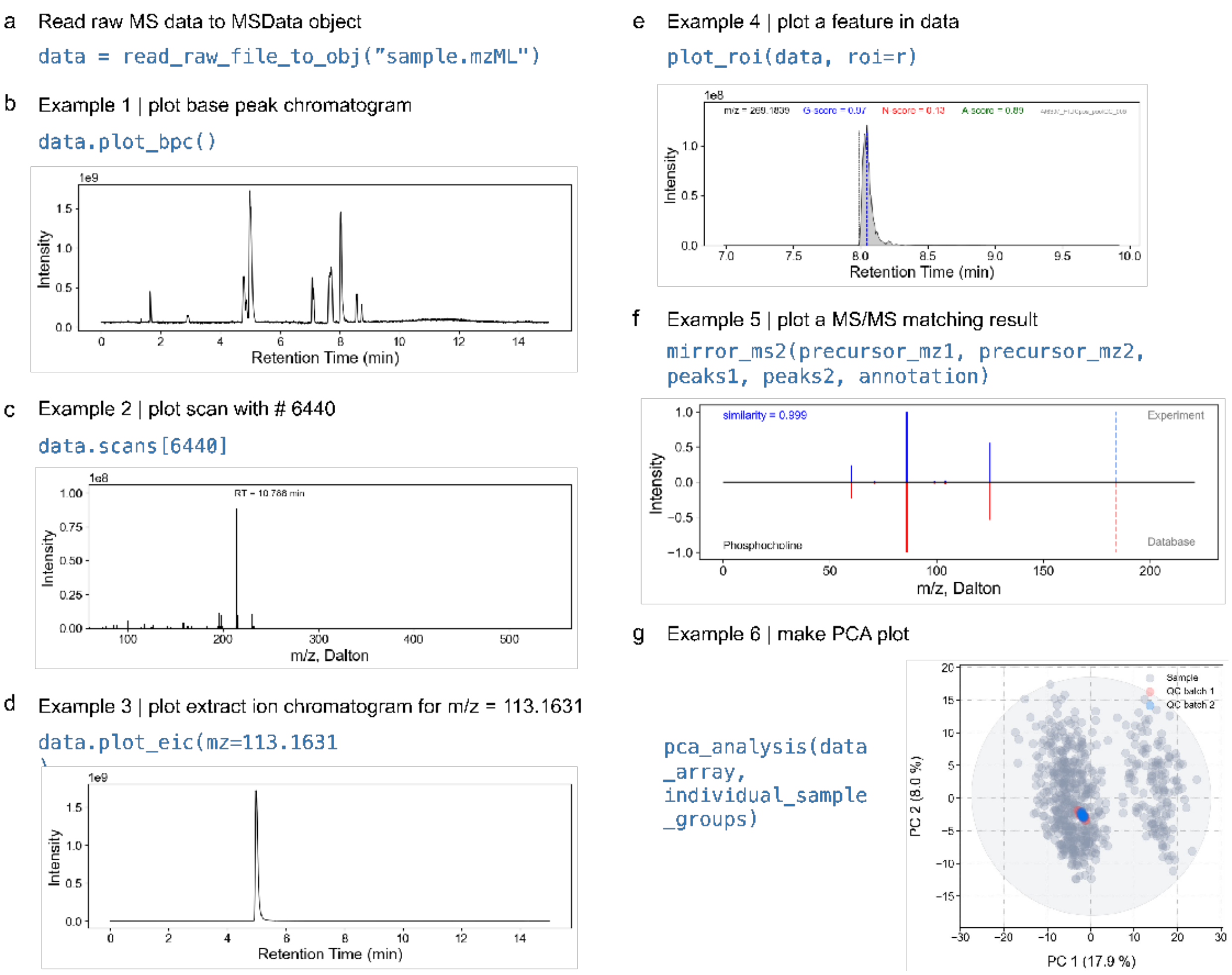

Supplementary Figure 9. Examples of data visualization in MassCube.

(a) Code block to read raw MS data in MassCube for visualization.

(b) Example for plotting a base peak chromatogram

(c) Example for plotting a single MS1 scan

(d) Example for plotting an extracted ion chromatogram across the entire chromatogram

(e) Example for plotting a detected peak

(f) Example for plotting a MS/MS matching result

(g) Example for plotting a principal component analysis plot

Code to generate the plots using the *masscube* Python package is shown in blue.

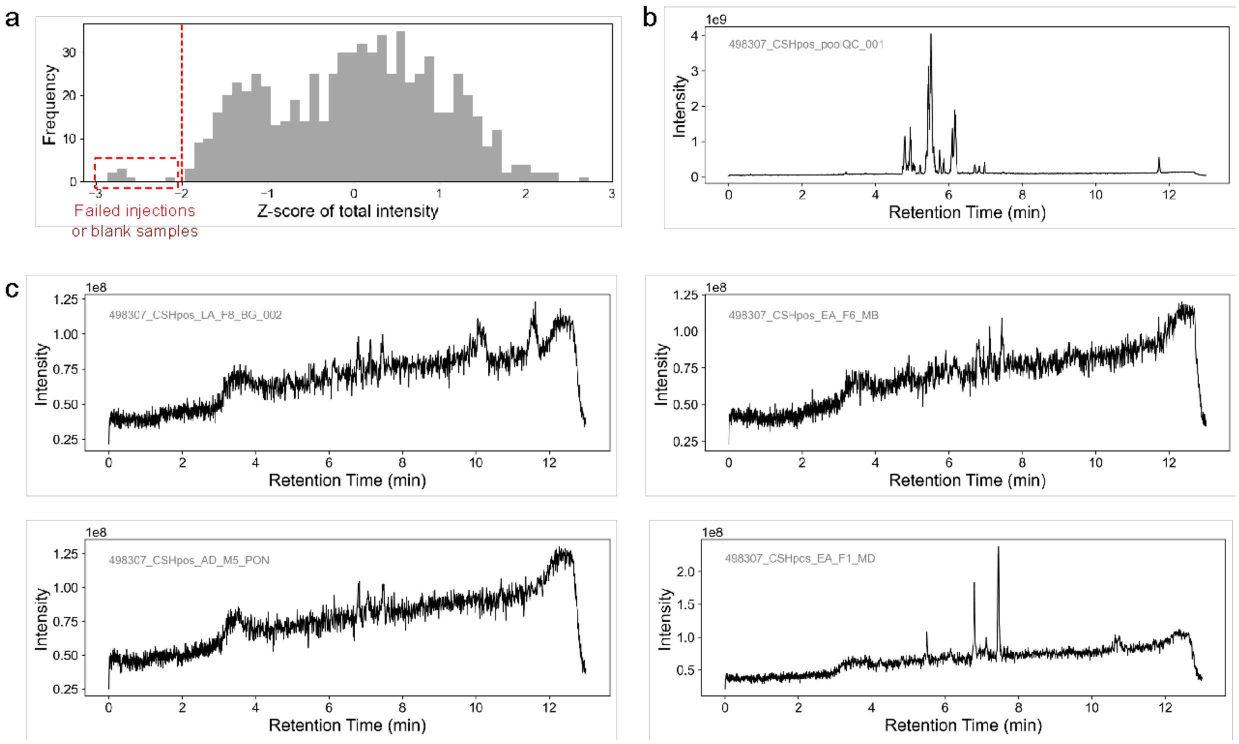

Supplementary Figure 10. **Detected failed sample injections by MassCube.**

(a) Distribution of Z-scores computed using total intensities from detected features.

(b) Base peak chromatogram of a normal sample injection.

(c) Base peak chromatograms of four detected failed sample injections. The lipidomics reverse phase positive mode data from the *Metabolome Atlas of the Aging Mouse Brain* dataset were used for demonstration.

Supplementary Note 1. Metadata management in MassCube

MassCube provides a diverse range of data processing functions and modules to support various application-oriented data processing tasks. Therefore, it is crucial to record all data processing steps in sequence, along with the parameters used, to ensure that the entire workflow can be traced and reproduced. We propose a stepwise framework to store the complete workflow, following the structure outlined below:

```
medadata = [
{
"name": "overview",
"layer": 0,
"packages": [
{"name": "masscube", "version": version("masscube")},
{"name": "numpy", "version": version("numpy")},
   …
],
"start_time": (time when data analysis begins)
…
},
{
"name": " feature_detection",
"layer ": 1,
(parameters): (value),
….
},
….
]
```

where the key "name" describes the name of each step, "layer" controls the order in which the steps are executed, and (parameters):(value) records the step-specific data processing parameters. This structured approach ensures clarity and reproducibility of the data processing workflow within MassCube.

Supplementary Note 2. LC-MS analysis of human plasma samples of Alzheimer's Disease patients

473 plasmas were analyzed by both Exploris 240 Orbitrap and Astral Orbitrap (Thermo Scientific, San Jose) from an Alzheimer's patients exposome cohort from Prof. Rima Kaddoura-Daouk at Duke University. Briefly, 20 µL of plasma was extracted by Matyash biphasic extraction. The lower aqueous phase was transferred to two aliquots which both were dried down and resuspended with 100 µL of ACN:Water (80:20) with isotope-labeled internal standards. One aliquot was analyzed by Exploris 240 and the other aliquot was analyzed by Astral. 3 µL of the polar metabolite extract was injected into HILIC-MS. The HILIC column was ACQUITY Premier BEH Amide Column, 1.7 µm, 2.1 mm x 50 mm. Mobile phases A and B were water and 95% ACN both with 10mM ammonium formate and 0.125% formic acid. The LC gradient started with 100% of B and decreased to 30% B from 0.5 to 2.55 min, followed by 3.15 to 3.8 min of equilibration back to 100% of B. The LC method was kept the same between Exploris 240 and Astral. The polar metabolites were analyzed in both positive and negative ionization modes. In Exploris 240, every sample was acquired by MS1 full scan (60-900 mass range, 60,000 mass resolution, 1e6 AGC target) and top-2 DDA MS2 (15,000 mass resolution, 10^5 AGC target, NCE 30-50-80%). 8 rounds of iterative exclusion were acquired on the pooled QC sample by automated Acquire X. In the Astral system, every sample was analyzed with full scan MS1 with Orbitrap analyzer (60-900 mass range, 60,000 mass resolution, 1e6 AGC target) and MS/MS scans with Astral analyzer (15,000 mass resolution, 1% of 1e5 AGC target, NCE of 40%). With the same 0.2 msec cycle time, the MS/MS by Astral with 1% of 1e5 AGC target was approximately equivalent to top-35 data-dependent acquisition. Pooled quality control, reference material NIST 1950 plasma, and blanks were acquired every 10 sample injections for quality control purposes.

Supplementary Note 3. Sample preparation workflow and experimental configurations for biological application.

Sample preparation

Lipids and polar metabolites were separated through biphasic solvent extraction for LC-MS/MS analysis. Five milligrams of tissue from each brain region were homogenized in 225 µL of −20 °C cold, internal standard-containing methanol using a GenoGrinder 2010 (SPEX SamplePrep) for 2 min at 1,350 rpm. The extraction methanol contained the following internal standards for quality control and retention time normalization: sphingosine (d17:1), LPE (17:1), LPC (17:0), MG (17:0/0:0/0:0), DG (12:0/12:0/0:0), PC (12:0/13:0), cholesterol-d_7, SM (18:1/17:1), ceramide (d18:1/17:0), PE (17:0/17:0), TG (14:0/16:1/14:0)-d_5, TG (17:0/17:1/17:0)-d_5, acylcarnitine (18:1)-d3, fatty acid (16:0)-d_3, MAG (17:0/0:0/0:0), PI (15:0–18:1)-d_7, PG (17:0/17:0), PS (15:0-18:1)-d_7, glucosylceramide(d18:1/17:0), mono-sulfo galactosylceramide(d18:1/17:0), and 5-PAHSA-d_9. The homogenate was vortexed for 10 s. 750 µL of −20 °C cold, internal standard-containing methyl tertiary-butyl ether (MTBE) was added, and the mixture was vortexed for 10 s and shaken at 4 °C for 5 min with an Orbital Mixing Chilling/Heating Plate (Torrey Pines Scientific Instruments). MTBE contained cholesteryl ester 22:1 as internal standard. Next, 188 µL room temperature water was added and vortexed for 20 s to induce phase separation. After centrifugation for 2 min at 14,000×g, two 350 µL aliquots of the upper non-polar phase and two 125 µL aliquots of the bottom polar phase were collected and dried down. The remaining fractions were combined to form QC pools and were injected after every set of 10 biological samples.

The non-polar phase employed for lipidomics was resuspended in a mixture of methanol/toluene (60 µL, 9:1, v/v) containing an internal standard [12-[(cyclohexylamine) carbonyl]amino]-dodecanoic acid (CUDA)] before injection. Resuspension of dried polar phases for HILIC analysis was performed in a mixture of acetonitrile/water (90 µL, 4:1, v/v) containing the following internal standards: CUDA, caffeine-d_9, acetylcholine-d_4, TMAO-d_9, 1-methylnicotinamide-d_3, Val-Tyr-Val, betaine-d_9, acyl carnitine (2:0)-d_3, N-methyl-histamine-d_3, l-carnitine-d_3, butyrobetaine-d_9, l-

glutamine-d_5, aspartic acid-d_3, l-arginine-$^{15}N_2$, cystine-d_4, asparagine-d_3, histidine-d_5, isoleucine-d_{10}, leucine-d_{10}, methionine-d_8, ornithine-d_2, phenylalanine-d_8, proline-d_7, threonine-d_5, tryptohan-d_8, tyrosine-d_7, valine-d_8, spermine-d_8, glucose-d_7, fructose-6-phosphate-$^{13}C_6$, succinic acid-d_4, taurocholic acid-d_4, adenosine 5′-monophosphate-$^{15}N_5$, uridine 5′-monophosphate-$^{15}N_2$, dopamine-d_4, taurine-d_4, uracil-d_2, biotin-d_4, N-acetylalanine-d_3, guanine-^{13}C, and adenosine-$^{13}C_5$.

Untargeted lipidomics analysis

For lipidomics analysis, 3 µL of the resuspended non-polar phase was injected into a Vanquish UHPLC system (Thermo Scientific, Waltham, MA, USA) equipped with a Waters Acquity UPLC CSH C18 (100 mm × 2.1 mm i.d.; 1.7 µm) coupled with a Waters Acquity VanGuard CSH C18 precolumn (5 mm × 2.1 mm i.d.; 1.7 µm). The oven temperature and flow rate were set at 65 °C and 0.6 mL/min, respectively. In order to obtain a broad lipid coverage, different mobile phases were employed for positive mode and negative mode analysis, respectively. The positive mobile phase consists of acetonitrile/water (60/40, v/v) with 0.1% formic acid and 10 mM ammonium formate as A and 2-propanol/acetonitrile (90:10, v/v) with 0.1% formic acid and 10 mM ammonium formate as B, while the negative mode mobile phase is made up of acetonitrile/water (60/40, v/v) with 10 mM ammonium acetate as A and 2-propanol/acetonitrile (90/10, v/v) with 10 mM ammonium acetate as B. Both modes share the same gradient: 0–2 min from 15% to 30% B, 2–2.5 min from 30% to 48% B, 2.5–11 min from 48% to 82% B, 11–11.5 min from 82% to 99% B, 11.5–12 min maintain at 99% B, 12–12.1 min from 99% to 15% B, and 12.1–14.2 min re-equilibrate at 15% B. A ThermoFisher Q-Exactive HF with a HESI-II ion source (Thermo Scientific, Waltham, MA, USA) was used to collect spectra with a data-dependent MS/MS spectra acquisition method. The ion source conditions were set as follows: spray voltage, 3.6 kV; sheath gas flow rate, 60 arbitrary units; aux gas flow rate, 25 arbitrary units; sweep gas flow rate, 2 arbitrary units; capillary temp, 300 °C; S-lens RF level, 50; Aux gas heater temperature, 370 °C. The following acquisition parameters were used for MS1 analysis: resolution, 60,000, AGC target,

1e6; Maximum IT, 100 ms; scan range 150–1700 m/z; spectrum data type, centroid. Data-dependent MS/MS parameters: resolution, 15,000; AGC target, 1e5; maximum IT, 50 ms; loop count, 4; TopN, 4; isolation window, 1.0m/z; fixed first mass, 70.0m/z; (N)CE/stepped nce, 20, 30, 40; spectrum data type, centroid; minimum AGC target, 8e3; intensity threshold, 1.6e5; exclude isotopes, on; dynamic exclusion, 3.0 s. To increase the total number of MS/MS spectra, five runs with iterative MS/MS exclusions were performed using the R package “IE-Omics”18 for both positive and negative electrospray conditions.

Untargeted metabolomics analysis

For LC–MS/MS analysis of polar metabolites, the same ThermoFisher equipment was used as above. Three microliters of the resuspended HILIC solution was injected onto a Waters Acquity UPLC BEH Amide column (150 mm × 2.1 mm; 1.7 µm) coupled with an additional Waters Acquity VanGuard BEH Amide precolumn (5 mm × 2.1 mm; 1.7 µm). The oven temperature was maintained at 45 °C, and the flow rate was set at 0.4 mL/min. HILIC chromatographic separations were performed by the following parameters: solvent A consisted of water with 10 mM ammonium formate and 0.125% formic acid, solvent B was made from acetonitrile/water (95/5, v/v) with 10 mM ammonium formate and 0.125% formic acid. A gradient run was set up as 0–2 min at 100% B, 2–7.7 min from 100% to 70% B, 7.7–9.5 min from 70% to 40% B, 9.5–10.25 min from 40% to 30% B, 10.25–12.75 min from 30% to 100% B, and 12.75–17 min re-equilibrate at 100% B. Mass spectrometry parameters were identical as above, but the MS1 mass was limited to 60–900 m/z.

Supplementary Note 4. Failed sample injection detection algorithm

Failed sample injections can occur for various reasons, including insufficient sample volume, uncalibrated needle position, and improper insert position in the vial. When an injection fails, the sample should not be used for downstream data normalization or statistical analysis. It can be anticipated that if a quality control sample fails, intensity normalization may be compromised due to extremely low intensity values in that sample. MassCube automatically evaluates the data and identifies failed sample injections using an outlier detection algorithm.

Individual data files were first processed in MassCube for feature detection. Using all the detected features, MassCube computed the total intensity (using peak height by default) for all the n files in a study, resulting in the set {Int_1, …Int_n}. Z-scores were subsequently calculated using the total intensity values, yielding {z_1, …, z_n}. A file i is considered an outlier when

$$z_i > zscore_{tol}$$

where the tolerance of Z-score was set to 2 by default. Importantly, blank samples, which typically exhibit significantly lower total intensity, are not considered as failed injections.

Supplementary Note 5. Outline for source code.

All source code for data processing was provided at https://zenodo.org/records/14159704. To ensure proper versioning and accessibility, we prepared an outline for all source code in Python and R, with input and output data.

1) 41_MetaboLights_data_peak_evaluation.zip
    a. Feature detection results for 200 raw single files
    b. CODE_feature_eval_public_data.ipynb: Python script for feature evaluation
2) Synthetic_data_benchmarking.zip
    a. CODE_synthetic_data_benchmarking.ipynb: Python script for generating synthetic data
    b. Inserted_shoulder_peaks.csv: list of inserted MS signals
    c. simulated_file.mzML: generated mzML file
3) Experimental_data_benchmarking.zip
    a. CODE_real_data_benchmarking.ipynb: Python script for benchmarking
    b. Raw data files
    c. All feature detection results from MassCube
    d. All feature detection results from MS-DIAL
    e. All feature detection results from MZmine3
    f. All feature detection results from *xcms*
4) Manually_labeled_EICs.zip
    a. double_peaks: all manually labeled double peaks
    b. single_peaks: all manually labeled single peaks
5) Mouse_brain_MassCube_output.zip
6) Mouse_brain_AD_MA_classification.zip

In addition, active development is ongoing for the MassCube project, aiming to provide users with comprehensive toolboxes for diverse, application-oriented data processing. Please follow our updates at https://huaxuyu.github.io/masscubedocs/docs/plans/.
