# Supplementary material for: MassCube improves accuracy for metabolomics data processing from raw files to phenotype classifiers
Source: Nat Commun. 2025 Jul 1;16:5487. doi: 10.1038/s41467-025-60640-5 (PMC12216001; doi:10.1038/s41467-025-60640-5)
Supplement: Supplementary file 2 — Reporting Summary [file 41467_2025_60640_MOESM2_ESM.pdf]

nature portfolio

Corresponding author(s): Oliver Fiehn

Last updated by author(s): May 21, 2025

Reporting Summary

Nature Portfolio wishes to improve the reproducibility of the work that we publish. This form provides structure for consistency and transparency in reporting. For further information on Nature Portfolio policies, see our Editorial Policies and the Editorial Policy Checklist.

Statistics

For all statistical analyses, confirm that the following items are present in the figure legend, table legend, main text, or Methods section.

n/a	Confirmed	
☐	☒	The exact sample size (n) for each experimental group/condition, given as a discrete number and unit of measurement
☐	☒	A statement on whether measurements were taken from distinct samples or whether the same sample was measured repeatedly
☐	☒	The statistical test(s) used AND whether they are one- or two-sided<br>*Only common tests should be described solely by name; describe more complex techniques in the Methods section.*
☒	☐	A description of all covariates tested
☐	☒	A description of any assumptions or corrections, such as tests of normality and adjustment for multiple comparisons
☐	☒	A full description of the statistical parameters including central tendency (e.g. means) or other basic estimates (e.g. regression coefficient) AND variation (e.g. standard deviation) or associated estimates of uncertainty (e.g. confidence intervals)
☐	☒	For null hypothesis testing, the test statistic (e.g. F, t, r) with confidence intervals, effect sizes, degrees of freedom and P value noted<br>*Give P values as exact values whenever suitable.*
☒	☐	For Bayesian analysis, information on the choice of priors and Markov chain Monte Carlo settings
☒	☐	For hierarchical and complex designs, identification of the appropriate level for tests and full reporting of outcomes
☐	☒	Estimates of effect sizes (e.g. Cohen's d, Pearson's r), indicating how they were calculated

Our web collection on statistics for biologists contains articles on many of the points above.

Software and code

Policy information about availability of computer code

Data collection	Eight experimental LC-MS/MS data were used for benchmarking feature detection performance, including NIST SRM 1950 plasma data, acquired on Thermo Orbitrap Exploris 480 MS, reverse phase (RP) positive ion mode; NIST SRM 1950 plasma data, acquired on Thermo Q Exactive MS, RP negative ion mode; mouse plasma data, acquired on Thermo Q Exactive HF MS, hydrophilic interaction chromatography (HILIC) positive ion mode; NIST Human Fecal Material RGTM 10162 data, acquired on Thermo Q Exactive MS, HILIC negative ion mode; Human serum data, acquired on Bruker impact II QTOF MS, RP positive ion mode; Mouse feces data, acquired on Bruker impact II QTOF MS, HILIC positive ion mode; whole fruit fly (D. melanogaster strains) data, acquired on Bruker impact II QTOF MS, HILIC positive ion mode; and human urine data, acquired on Bruker impact II QTOF MS, HILIC negative ion mode. The raw MS data files for the Atlas of the Aging Mouse Brain were obtained from a previous study (https://www.nature.com/articles/s41467-021-26310-y), acquired using a ThermoFisher Q-Exactive HF with a HESI-II ion source (Thermo Scientific, Waltham, MA, USA) coupled with a Vanquish UHPLC system (Thermo Scientific, Waltham, MA, USA).
Data analysis	Source code for MassCube is available at https://github.com/huaxuyu/masscube. MassCube 1.0.22, MS-DIAL 4.9, MZmine 3.90, and xcms R package 4.0.0 were all used to process the simulated and experimental data for benchmarking. UMAP visualization was conducted in Python using the umap package. Chemical classification was performed using the ClassyFire Batch Compound Classification tool (https://cfb.fiehnlab.ucdavis.edu). Chemical Similarity Enrichment Analysis was conducted using the ChemRICH tool (https://chemrich.fiehnlab.ucdavis.edu/). Python and R code for data processing are available at https://zenodo.org/records/14159704.

For manuscripts utilizing custom algorithms or software that are central to the research but not yet described in published literature, software must be made available to editors and reviewers. We strongly encourage code deposition in a community repository (e.g. GitHub). See the Nature Portfolio guidelines for submitting code & software for further information.

Data

Policy information about availability of data

All manuscripts must include a data availability statement. This statement should provide the following information, where applicable:

- Accession codes, unique identifiers, or web links for publicly available datasets
- A description of any restrictions on data availability
- For clinical datasets or third party data, please ensure that the statement adheres to our policy

Synthetic MS data used for algorithm benchmarking are available at https://zenodo.org/records/14159704 including the complete list of inserted MS signals. Eight raw experimental LC-MS data files for software benchmarking in Fig. 3 are provided in https://zenodo.org/records/14159704. The access to human plasma samples of Alzheimer's Disease patients collected on both Orbitrap Exploris 240 and Orbitrap Astral MS are on demand. Four LC-MS datasets used for speed benchmarking are available from the MassIVE and MetaboLights repositories: Orbitrap #1: Orbitrap data of NIST Human Fecal Material Standards, MSV000086988; Orbitrap #2: Orbitrap data of urine metabolomics storage study, MSV000091929; QTOF #1: QTOF data of plant metabolomics, MTBLS188; QTOF #2: QTOF data of Type 1 diabetes plasma lipidomics study, MTBLS620). 41 public datasets used for modeling the distribution of peak metadata were obtained from MetaboLights, with their study identifiers provided at https://zenodo.org/records/14159704. Data of the Atlas of the Aging Mouse Brain can be accessed from the Metabolomics Workbench under Project ID PR001047. Data from the NIST human fecal material standards are available from the MassIVE repository under MSV000086989. The NIST23 Tandem Mass Spectral Library used in biological application can be obtained from NIST. Supplementary Data Files including raw output from MassCube by re-analyzing the data of the Atlas of the Aging Mouse Brain, eight experimental LC-MS data files used for algorithm benchmarking, EICs in .png format for manually labeled experimental data for algorithm benchmarking, MassCube's ouput of 41 metabolomics studies including 200 individual files from MetaboLights and MassCube's output of the phenotype classifier for mouse brain data are available at https://zenodo.org/records/14159704 .

Research involving human participants, their data, or biological material

Policy information about studies with human participants or human data. See also policy information about sex, gender (identity/presentation), and sexual orientation and race, ethnicity and racism.

Reporting on sex and gender	No sex or gender information was used in this study. The dataset was solely intended for speed benchmarking and software stability demonstration.
Reporting on race, ethnicity, or other socially relevant groupings	n/a
Population characteristics	n/a
Recruitment	n/a
Ethics oversight	The Alzheimer's disease dataset used for speed benchmarking is an exploratory study coordinated by Duke University under R. Kaddurah-Daouk. The protocol was approved by the UC San Diego's Institutional Review Board (IRB) protocol #202063, Indiana University IRB study #1011003338, Kansas University IRB study #CR00020412, University of Wisconsin IRB study IORG0000056 approved 3-29-2023, New York University IRB study #i20-00427. Written informed consent was obtained from all participants.

Note that full information on the approval of the study protocol must also be provided in the manuscript.

Field-specific reporting

Please select the one below that is the best fit for your research. If you are not sure, read the appropriate sections before making your selection.

[x] Life sciences [ ] Behavioural & social sciences [ ] Ecological, evolutionary & environmental sciences

For a reference copy of the document with all sections, see nature.com/documents/nr-reporting-summary-flat.pdf

Life sciences study design

All studies must disclose on these points even when the disclosure is negative.

Sample size	Biological application was performed by reanalyzing a dataset from a previously published study (the Atlas of the Aging Mouse Brain, https://www.nature.com/articles/s41467-021-26310-y), which encompassed a total of 702 mouse brain samples across 80 sample groups representing ten regions (cerebral cortex, olfactory bulb, hippocampus, hypothalamus, basal ganglia, thalamus, midbrain, pons, medulla and cerebellum), two sexes (male and female), and four age groups (adolescence, 3 weeks of age; early adulthood, 16 weeks of age; middle-age, 59 weeks of age; old age, 92 weeks).
Data exclusions	No exclusion.
Replication	Each biological group contains eight biological replicates. The analytical reproducibility of the mass spectrometry was verified using quality control samples, which was periodically injected after every 10 biological samples.
Randomization	Samples from different biological groups were randomize for LC-MS analysis.

Blinding	Blinding of results were no necessary as the data were obtained from the previous study for re-analysis.

Reporting for specific materials, systems and methods

We require information from authors about some types of materials, experimental systems and methods used in many studies. Here, indicate whether each material, system or method listed is relevant to your study. If you are not sure if a list item applies to your research, read the appropriate section before selecting a response.

Materials & experimental systems

n/a	Involved in the study
☒	☐ Antibodies
☒	☐ Eukaryotic cell lines
☒	☐ Palaeontology and archaeology
☐	☒ Animals and other organisms
☒	☐ Clinical data
☒	☐ Dual use research of concern
☒	☐ Plants

Methods

n/a	Involved in the study
☒	☐ ChIP-seq
☒	☐ Flow cytometry
☒	☐ MRI-based neuroimaging

Animals and other research organisms

Policy information about studies involving animals; ARRIVE guidelines recommended for reporting animal research, and Sex and Gender in Research

Laboratory animals	Brain tissue samples were collected from 3, 16, 59, and 92 weeks old male and female wild-type mice on a C57BL/6N background. Mice were cohoused by gender groups of 4–5 in individually ventilated cages (Optimice IVC, Animal Care Systems, Centennial, CO) on a 12:12-h (6:00/18:00) light:dark cycle at 68–79°F with 40–60% humidity and provided water and standard rodent chow (Rodent chow, Harlan 2918) ad libitum.
Wild animals	n/a
Reporting on sex	The mouse study examined cohorts of 8 male and 8 female wild-type mice at four life stages including adolescence (AD, 3 weeks), early adulthood (EA, 16 weeks), middle age (MA, 59 weeks), and old age (OA, 92 weeks). Sex-based analysis was performed to investigate the metabolome and lipidome difference bewtween male and female mouse brain regions.
Field-collected samples	n/a
Ethics oversight	All procedures were approved by the IACUC of the University of California, Davis, which is an AAALAC-accredited institution. Animal housing and euthanasia were performed in accordance with the recommendations of the Guide for the Care and Use of Laboratory Animals.

Note that full information on the approval of the study protocol must also be provided in the manuscript.

Plants

Seed stocks	n/a
Novel plant genotypes	n/a
Authentication	n/a
